# Supplementary material for: Improved gRNA secondary structures allow editing of target sites resistant to CRISPR-Cas9 cleavage
Source: Nat Commun. 2022 Jan 25;13:489. doi: 10.1038/s41467-022-28137-7 (PMC8789806; doi:10.1038/s41467-022-28137-7)
Supplement: Supplementary file 1 — Supplementary Information [file 41467_2022_28137_MOESM1_ESM.pdf]

Supplementary Information

Improved gRNA secondary structures allow editing of target sites resistant to CRISPR-Cas9 cleavage

Riesenberg et al.

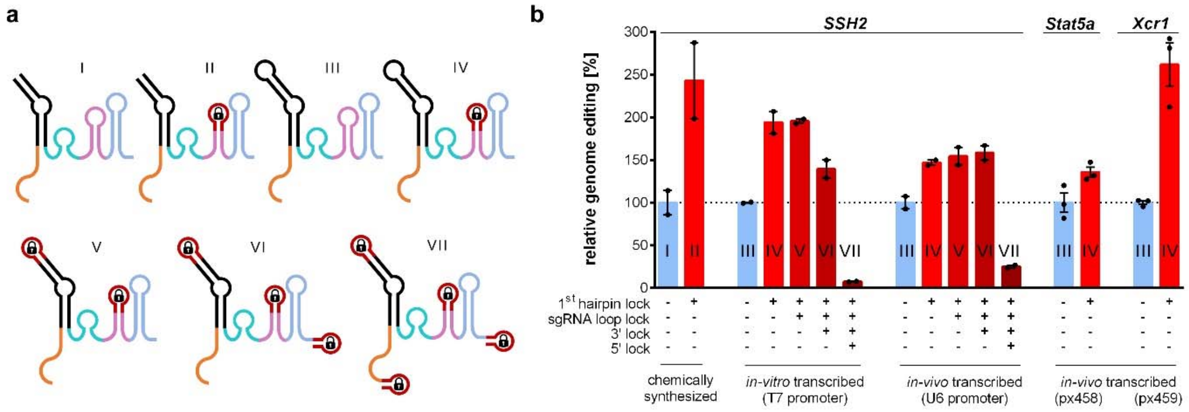

Supplementary Figure 1: Genome editing efficiencies of gRNAs carrying locked hairpins at different positions. (**a**) Design sketches of the gRNAs used in A labelled with roman numerals. Highlighted are the position of the locked hairpin (dark red), spacer sequence (orange), nexus (cyan), 1st hairpin (pink), and 2nd hairpin (blue). (**b**) Relative genome editing efficiencies of chemically synthesized, *in vitro* transcribed, and *in vivo* transcribed gRNAs that carry locked hairpins at different positions. Independent biological replicates (n = 2 for *SSH2*, n = 3 for *Stat5a* and *Xcr1*) are depicted by black dots and the error bars show the SEM. Oligonucleotides were electroporated into 409-B2 iCRISPR hiPSCs for *SSH2* or into SNL 76/7 mouse embryonic fibroblasts for *Stat5a* and *Xcr1*. For the plasmids px458 (Cas9-2A-GFP) and px459 (Cas9-2A-*Puro*) Cas9 is expressed by a CBh promoter while the gRNA is expressed from a U6 promoter. Cells electroporated with px459 were treated with 2µg/ml puromycin for two days starting one day post electroporation. Source data are provided as a Source Data file.

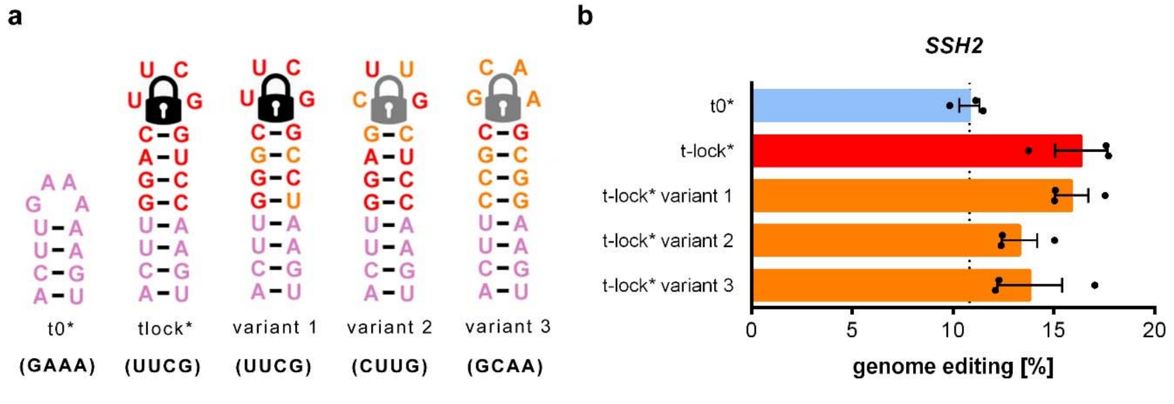

Supplementary Figure 2: Genome editing efficiencies of gRNAs with different locked hairpin variants. (**a**) 1st hairpin sequences of the normal tracrRNA (t0), locked tracrRNA (t-lock), and three different locked tracrRNA variants are shown. The elongated locked hairpin is colored red and nucleotide changes in the variants with superstable loops (UUCG, CUUG, GCAA) are orange. (**b**) Genome editing efficiencies of the crRNA:tracrRNA hybrid gRNAs for the SSH2 target. Independent biological replicates are depicted by black dots (n = 3) and the error bars show the SEM. The chemically synthesized tracrRNAs additionally have two phosphorothioate bonds at both the 3' and 5' terminus (*) to allow reasonable protection against degradation by nucleases. Oligonucleotides were electroporated into 409-B2 iCRISPR hiPSCs. Source data are provided as a Source Data file.

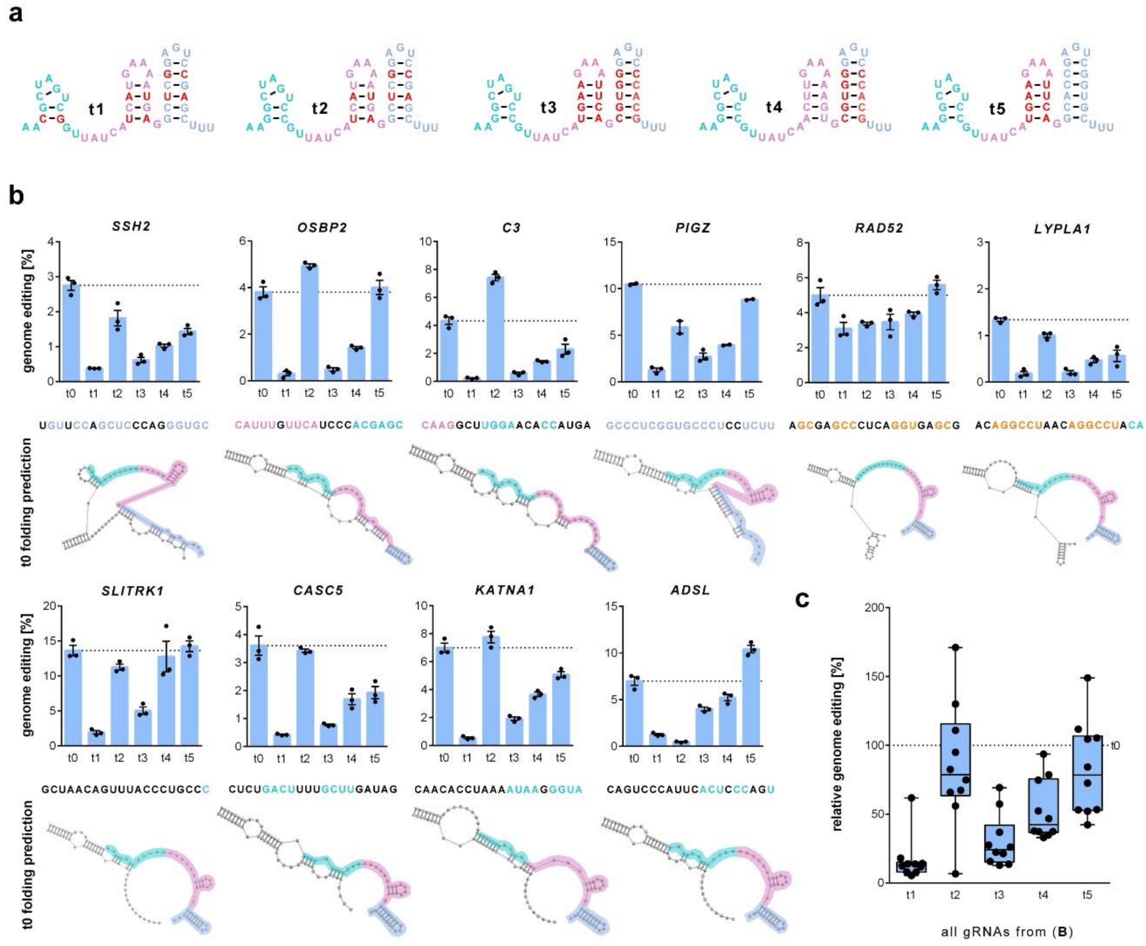

Supplementary Figure 3: Genome editing efficiencies of gRNAs with swaps of opposing bases. (**a**) The nexus, 1st hairpin and 2nd hairpin of the different base swap modifications (t1 to t5) are shown. The respective base swaps are colored red. (**b**) Genome editing efficiencies for t0 (normal) and the different swap designs t1 to t5. Independent biological replicates are depicted by black dots (n = 3, except *PIGZ* t0, t2, t4, t5 n = 2) and the error bars show the SEM. The panels show the efficiencies for 10 different gRNAs with different predicted non-canonical gRNA interactions to the nexus (cyan), 1st hairpin (pink), 2nd hairpin (blue), and/or the spacer sequence itself (orange). The predicted crRNA:tracrRNA structure from the *Bellaousov et al.* [1] RNAstructure web server is shown below the spacer sequence. (**c**) Box plots of relative genome editing efficiencies for all gRNAs from b (n = 10) with respect to the corresponding t0 tracrRNA set to 100% are shown. Boxes extend from the 25th to 75th percentile and show the median as a line. Whiskers extend from the minimum to maximum value. Chemically synthesized gRNAs (hybridized crRNA:tracrRNA) were lipofected into Cas9 expressing 409-B2 iCRISPR human induced pluripotent stem cells (hiPSCs). Source data are provided as a Source Data file.

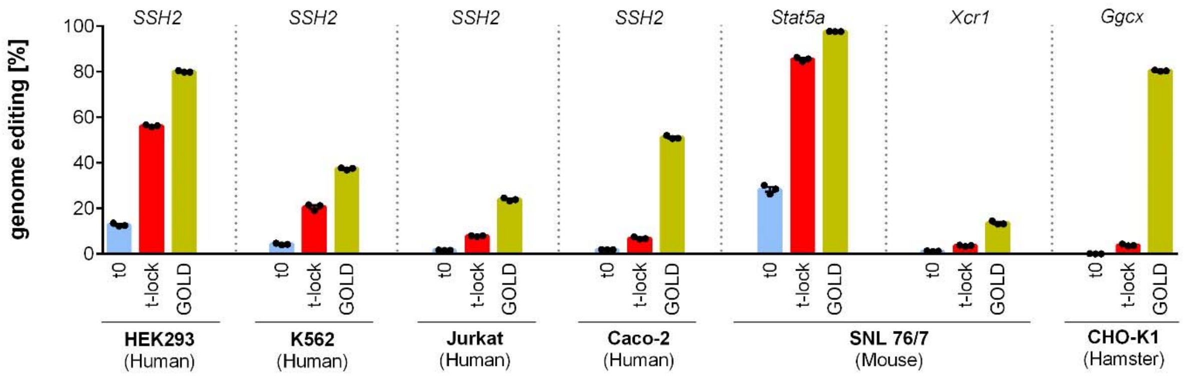

Supplementary Figure 4: Genome editing efficiencies of gRNAs with modified backbones in non-pluripotent cell types. Genome editing efficiency of normal tracrRNA (blue), t-lock tracrRNA (red), and GOLD tracrRNA (gold) hybridized with different crRNAs after electroporation of Cas9 RNP into different cell lines derived from human, mouse, and chinese hamster: human embryonic kidney 293 cells (HEK293), human myelogenous leukemia cells (K562), human T lymphocyte cells (Jurkat), human colorectal adenocarcinoma cells (Caco-2), mouse embryonic fibroblast derived cells (SNL 76/7), chinese hamster ovary cells (CHO-K1). Independent biological replicates are depicted by black dots (n = 3) and the error bars show the SEM. Source data are provided as a Source Data file.

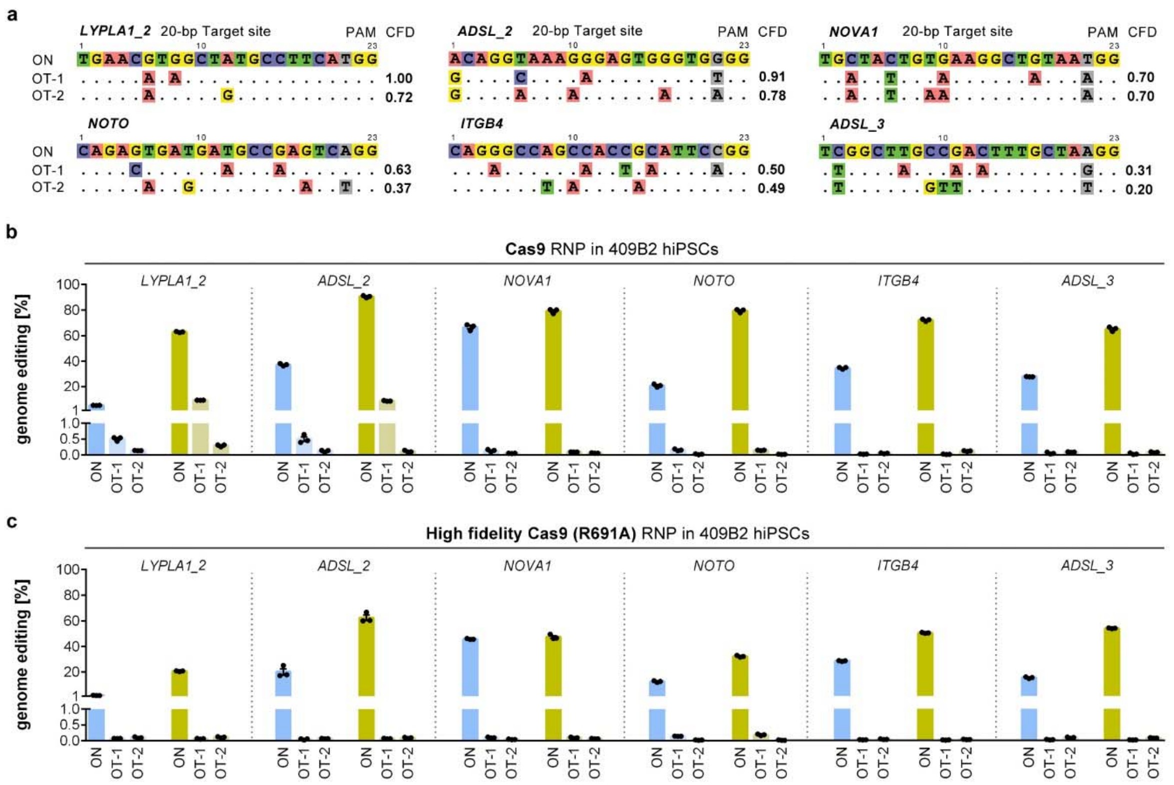

Supplementary Figure 5: Impact of optimized gRNA backbones on off-target editing. (**a**) On-target site sequence (ON) and top two CFD-score [2,3] predicted off-target site sequences (OT-1, OT-2) of six different gRNA spacers that have varying predicted tendency to generate off-targets. Non-mismatched bases are visualized by a dot. The CFD off-target scores are stated (ranging from 0 to 1; 1 = maximum off-target editing probability). (**b**) Genome editing efficiency of on-target and off-target sites for gRNAs from A when the normal (t0, in blue) or GOLD (in gold) tracrRNA are used for editing with Cas9. (**c**) Genome editing efficiency of on-target and off-target sites for gRNAs from A when the normal (t0) or GOLD tracrRNA are used for editing with the high fidelity Cas9 R691A variant (Cas9 HiFi). Cas9 RNPs were electroporated into 409-B2 hiPSCs. Independent biological replicates are depicted by black dots (n = 3) and the error bars show the SEM. Source data are provided as a Source Data file.

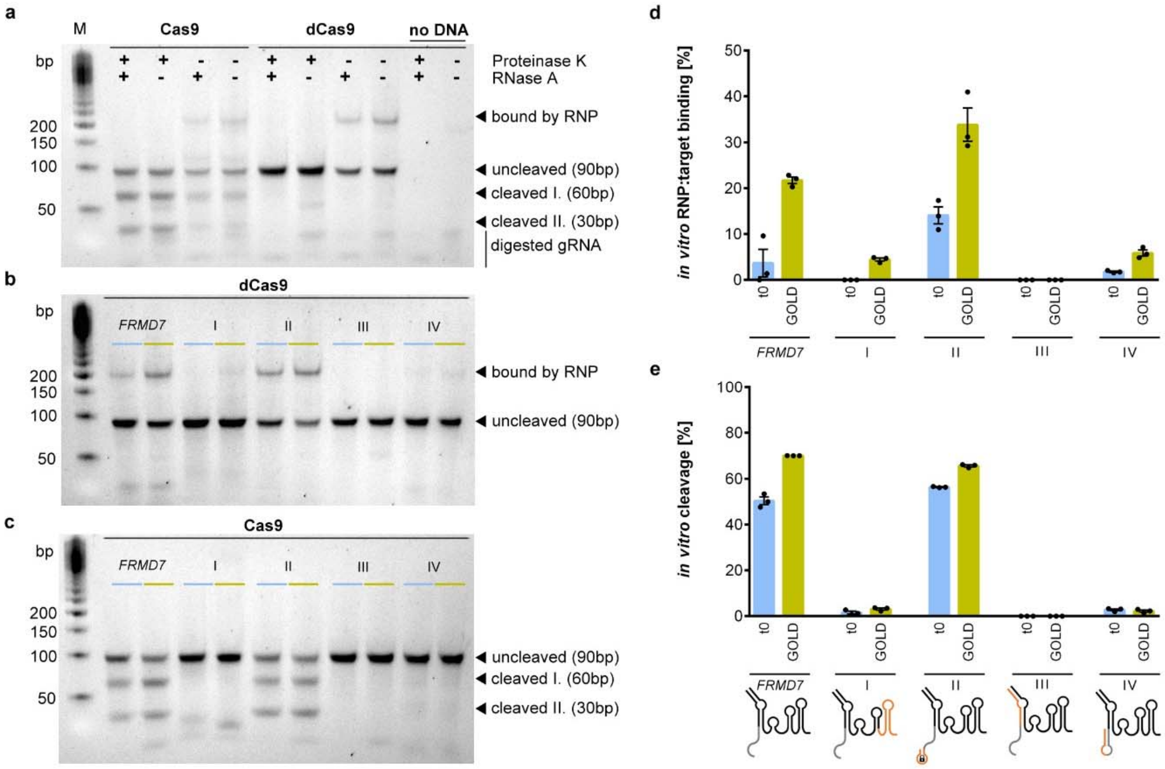

Supplementary Fig. 6: Impact of optimized gRNA backbones on *in vitro* binding and cleavage. (**a**) Exemplary 4% agarose gel of *FRMD7* crRNA:GOLD-tracrRNA incubated with 90bp dsDNA, that contains the target site and adjacent genomic sequence, and Cas9 or dead (d)Cas9 for 16h at 37°C. Columns show different subsequent treatments with Proteinase K and/or RNase and include no DNA controls. Blue light excitation of SYBR Gold II bound to double-stranded oligonucleotides results in visualization of separated bands corresponding to DNA bound by RNP, unbound and uncleaved DNA (90bp), as well as cleaved DNA (60bp and 30bp). Partially double-stranded gRNA can also result in faint bands. (**b**) Exemplary gel of dCas9 incubated with *FRMD7* and 'worst-case' crRNAs (I to IV) hybridized to either normal tracrRNA t0 (blue) or GOLD-tracrRNA (gold) and respective target DNA. Samples were not treated with Proteinase K or RNase A. (**c**) Exemplary gel of Cas9 incubated with *FRMD7* and 'worst-case' crRNAs (I to IV) hybridized to either normal tracrRNA to (blue) or GOLD-tracrRNA (gold) and respective target DNA. Samples were treated with both Proteinase K and RNase A. (**d**) High sensitivity DNA chip quantification of *in vitro* RNP:target binding of samples treated identically to those shown in b. (**e**) High sensitivity DNA chip quantification of *in vitro* cleavage of samples treated identically to those shown in c. Replicates from independent experiments are depicted by black dots (n = 3) and error bars show the SEM. Source data are provided as a Source Data file.

Supplementary information references

1 Bellaousov, S., Reuter, J. S., Seetin, M. G. & Mathews, D. H. RNAstructure: Web servers for RNA secondary structure prediction and analysis. *Nucleic Acids Res* **41**, W471-474, doi:10.1093/nar/gkt290 (2013).

2 Doench, J. G. *et al.* Optimized sgRNA design to maximize activity and minimize off-target effects of CRISPR-Cas9. *Nat Biotechnol* **34**, 184-191, doi:10.1038/nbt.3437 (2016).

3 Haeussler, M. *et al.* Evaluation of off-target and on-target scoring algorithms and integration into the guide RNA selection tool CRISPOR. *Genome Biol* **17**, 148, doi:10.1186/s13059-016-1012-2 (2016).
