# Supplementary material for: Improved gRNA secondary structures allow editing of target sites resistant to CRISPR-Cas9 cleavage
Source: Nat Commun. 2022 Jan 25;13:489. doi: 10.1038/s41467-022-28137-7 (PMC8789806; doi:10.1038/s41467-022-28137-7)
Supplement: Supplementary file 4 — Description of Additional Supplementary Files [file 41467_2022_28137_MOESM4_ESM.pdf]

Title: Supplementary Data 1:
Description: Oligonucleotides used in this study. Both RNA oligonucleotides (tracrRNA and crRNA spacer sequence) as well as DNA oligonucleotides (primer, other ssDNA and dsDNA) are shown. Chemical modifications used are: *=phosphorothioate bond, mN=2'OMe.
